# Soft tissue artifact compensation in lower extremities using displacement relationship between anatomical landmarks and skin markers

**DOI:** 10.1186/1757-1146-7-S1-A71

**Published:** 2014-04-08

**Authors:** Taebeum Ryu, Moonsoo Shin

**Affiliations:** 1 Department of Industrial and Management Engineering, Hanbat National University, Yusung, Daejeon, 305-719, South Korea

## 

Soft tissue artifact (STA), the deformation of skin and muscle during motion, is known to be one of the important sources of errors in human motion analysis using stereophotogrammetry. As a way to reduce the STA errors, methods estimating positions of anatomical landmarks (AL) have been proposed that keep them rigidly related to the underlying bone. The previous methods [1,2] used intermediate variables such as joint angles or motion time to adjust AL positions which were calculated from position data of skin markers. The present study proposes a method to estimate AL positions with skin marker positions directly, thereby removing the intermediate variables of the previous methods. The proposed method identifies a systematic relationship between the displacements of ALs and skin markers relative to a technical coordinate frame defined by skin markers in ad hoc motions(Figure 1). Then, AL positions are calibrated directly by using the displacement relationship with skin markers. The proposed method was applied to analyze three lower extremity motions (walking, sit-to-stand/stand-to-sit and step up/down) of ten healthy males. Its performance was compared with the transformation error minimization method (TEM) of [3] and the AL estimation method with joint angle (ALJ) of [2]. The proposed method considerably reduced STA errors relative to the TEM (by 30 – 80% ) and was also slightly more effective than the ALJ, showing 25 – 40% error reductions for seven of 18 kinematic variables(Figure 2).

**Figure 1:**
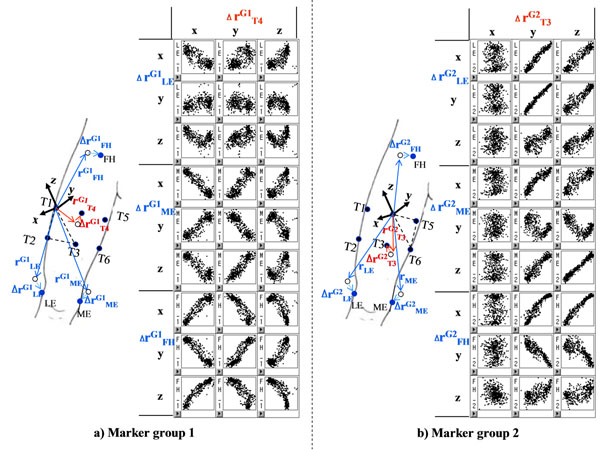
Scatter plot of AL and skin marker displacement in the thigh of a participant

**Figure 2:**
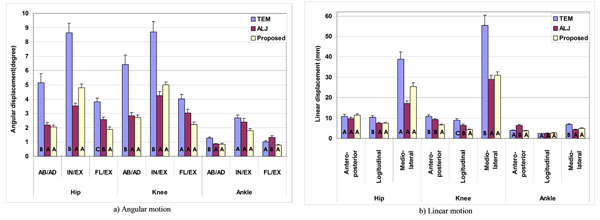
Statistical comparison of compensation methods

## Trial registration

Current Controlled Trials ISCRTN73824458.

## References

[B1] Cappello A Cappozzo A Palombara PFL Lucchetti L Leardini A Multiple anatomical landmark calibration for optimal bone pose estimation Human Movement Science 1997 16 259 274 10.1016/S0167-9457(96)00055-3

[B2] Lucchetti L Cappozo A Cappello A Croce UD Skin movement artefact assessment and compensation in the estimation of knee-joint kinematics Journal of Biomechanics 1998 31 977 984 10.1016/S0021-9290(98)00083-9 9880054

[B3] Soderkvist I Wedin P Determining the movements of the skeleton using well-configured markers Journal of Biomechanics 1993 26 1473 1477 10.1016/0021-9290(93)90098-Y 8308052

